# Spatial-Temporal Distribution of Hantavirus Rodent-Borne Infection by *Oligoryzomys fulvescens* in the Agua Buena Region - Panama

**DOI:** 10.1371/journal.pntd.0004460

**Published:** 2016-02-19

**Authors:** Blas Armién, Paulo Lazaro Ortiz, Publio Gonzalez, Alberto Cumbrera, Alina Rivero, Mario Avila, Aníbal G. Armién, Frederick Koster, Gregory Glass

**Affiliations:** 1 Department of Research in Emerging and Zoonotic Infectious Diseases, Gorgas Memorial Institute for Health Studies, Panama City, Panama; 2 Research Direction, Universidad Interamericana de Panama, Panama City, Panama; 3 Climate Center, Meteorology Institute, Havana, Cuba; 4 Ministry of Health, Panama City, Panama; 5 Department of Veterinary Population Medicine, CVM, University of Minnesota, Twin Cities, Minnesota, United States of America; 6 Lovelace Respiratory Research Institute, Albuquerque, New Mexico, United States of America; 7 The Emerging Pathogens Institute and Department of Geography, University of Florida, Gainesville, Florida, United States of America; Armed Forces Health Surveillance Center, UNITED STATES

## Abstract

**Background:**

Hotspot detection and characterization has played an increasing role in understanding the maintenance and transmission of zoonotic pathogens. Identifying the specific environmental factors (or their correlates) that influence reservoir host abundance help increase understanding of how pathogens are maintained in natural systems and are crucial to identifying disease risk. However, most recent studies are performed at macro-scale and describe broad temporal patterns of population abundances. Few have been conducted at a microscale over short time periods that better capture the dynamical patterns of key populations. These finer resolution studies may better define the likelihood of local pathogen persistence. This study characterizes the landscape distribution and spatio-temporal dynamics of *Oligoryzomys fulvescens* (*O*. *fulvescens*), an important mammalian reservoir in Central America.

**Methods:**

Information collected in a longitudinal study of rodent populations in the community of Agua Buena in Tonosí, Panama, between April 2006 and December 2009 was analyzed using non-spatial analyses (box plots) and explicit spatial statistical tests (correlograms, SADIE and LISA). A 90 node grid was built (raster format) to design a base map. The area between the nodes was 0.09 km^2^ and the total study area was 6.43 km^2^ (2.39 x 2.69 km). The temporal assessment dataset was divided into four periods for each year studied: the dry season, rainy season, and two months-long transitions between seasons (the months of April and December).

**Results:**

There were heterogeneous patterns in the population densities and degrees of dispersion of *O*. *fulvescens* that varied across seasons and among years. The species typically was locally absent during the late transitional months of the season, and re-established locally in subsequent years. These populations re-occurred in the same area during the first three years but subsequently re-established further south in the final year of the study. Spatial autocorrelation analyses indicated local populations encompassed approximately 300–600 m. The borders between suitable and unsuitable habitats were sharply demarcated over short distances.

**Conclusion:**

*Oligoryzomys fulvescens* showed a well-defined spatial pattern that evolved over time, and led to a pattern of changing aggregation. Thus, hot spots of abundance showed a general shifting pattern that helps explain the intermittent risk from pathogens transmitted by this species. This variation was associated with seasonality, as well as anthropogenic pressures that occurred with agricultural activities. These factors help define the characteristics of the occurrence, timing, intensity and duration of synanthropic populations affected by human populations and, consequently, possible exposure that local human populations experience.

## Author Summary

In Panama, hantavirus pulmonary syndrome (HPS) is due to infection with the Choclo virus, which is transmitted by the rice rat (*Oligoryzomys fulvescens*). Hantavirus infection is endemic in rural, Central-West Panama, an area characterized by a very dynamic combination of subsistence and industrial agriculture. This region contains one of the highest hantavirus antibody prevalence (16–62%) in humans on the continent, but with low mortality (17%) in the affected population. People become infected in and around the household area. Evidence points to the increase in and dynamic nature of agricultural activity in this region as the primary causes of increased hantavirus infection in humans in Panama. This study was conducted on a high spatio-temporal resolution that better captures how rapidly the abundances of reservoir populations shift—altering this aspect of zoonotic spillover of pathogens. Practically, the results demonstrate an important challenge in monitoring or predicting zoonotic disease outbreaks at the level of reservoir populations. These populations are subject to fluctuations over time (both within and among years). The populations themselves are geographically restricted, even within their broader range. Suitable habitats for rodents may change but are poorly understood. Our results are valuable to understand hantavirus dynamics in Panama and the many other countries from North to South America in which hantaviruses are endemic in host populations.

## Introduction

Outbreaks of zoonotic diseases in human and domestic animal populations often occur with little warning as pathogens spillover from wildlife populations. These events are associated with specific habitats and are often presaged by changes in local environmental conditions. For example, Tucker et al., (2002) identified an environmental signal associated with historical outbreaks of Ebola virus in western Africa in 1994–1996 [1]. However, despite the presumption that hotspots for zoonotic agents occur in the environment, relatively little is understood about the sporadic occurrence of local reservoir populations, the duration and spatial relationships (size and connectedness) of suitable habitats for reservoirs, or the relationship between host populations and the environment. For example, Glass et al., (2007) reported that only a very small portion of the environment in a study site in the southwestern United States was consistently suitable for habitation by deer mice potentially infected with Sin Nombre virus [2]. Suitable habitats typically persisted for only a year or two, and most local reservoir populations were ephemeral. This temporal dynamism of host populations may play a significant role in the risk associated with pathogens such as hantavirus. Rodent-borne zoonoses are likely sensitive to reservoir population size, demographic characteristics, and population persistence; these factors in conjunction with temporal changes in host population distribution are important determinants of pathogen establishment and local intensity of transmission within host populations. This intensity plays a role in the subsequent spillover of the pathogen from its maintenance cycle into other species [3].

Prior to the recent Ebola outbreak, the identification of hantaviral diseases in North America provided one of the best studied examples of such an emergence [4,5]. Since the initial outbreak of HPS in 1993, human cases of HPS have occurred sporadically within North America. While the broad geographic region of North America at greatest risk is well known, the specific localities where the disease re-emerges are poorly anticipated. Several longitudinal studies showed that local reservoir populations were often subject to extinction and the viruses appeared only sporadically—often being absent [6,7]. Although it is assumed that local persistence is a function of finite population size, host persistence, and transmission dynamics, the details of the local dynamics of rodent hosts have been rarely considered.

Currently, 17 hantaviruses that produce pulmonary disease in humans have been identified [4,8]. One of them is the Choclo virus, which was first detected in 2000 [9]. In Panama, *Oligoryzomys fulvescens* is the reservoir host species [9,10]. Studies indicate that the primary hantavirus outbreak in the Central-West region of Panama was due to anthropogenic disturbance of the ecosystem, associated with intense agricultural activity, as well as possible environmental factors (i.e., increased precipitation) [10] with people at most risk in and around their homes [11]. Similar observations have been reported from Paraguay and Brazil [4]. In the Azuero peninsula, the prevalence of hantavirus IgG antibodies in *O*. *fulvescens* may range between 18 and 29% [11]. Spatial-temporal analyses of the *O*. *fulvescens*’ home range and movement, which may define a potential area for of risk of infection and transmission of Choclo virus, have not been conducted. Here, we examine the pattern, dissemination, and the spatial and temporal distribution of *O*. *fulvescens*. In addition, we explore the potential ecological processes that drive the patterns and heterogeneity in this region.

## Methods

### Study area

**Fig 1:**
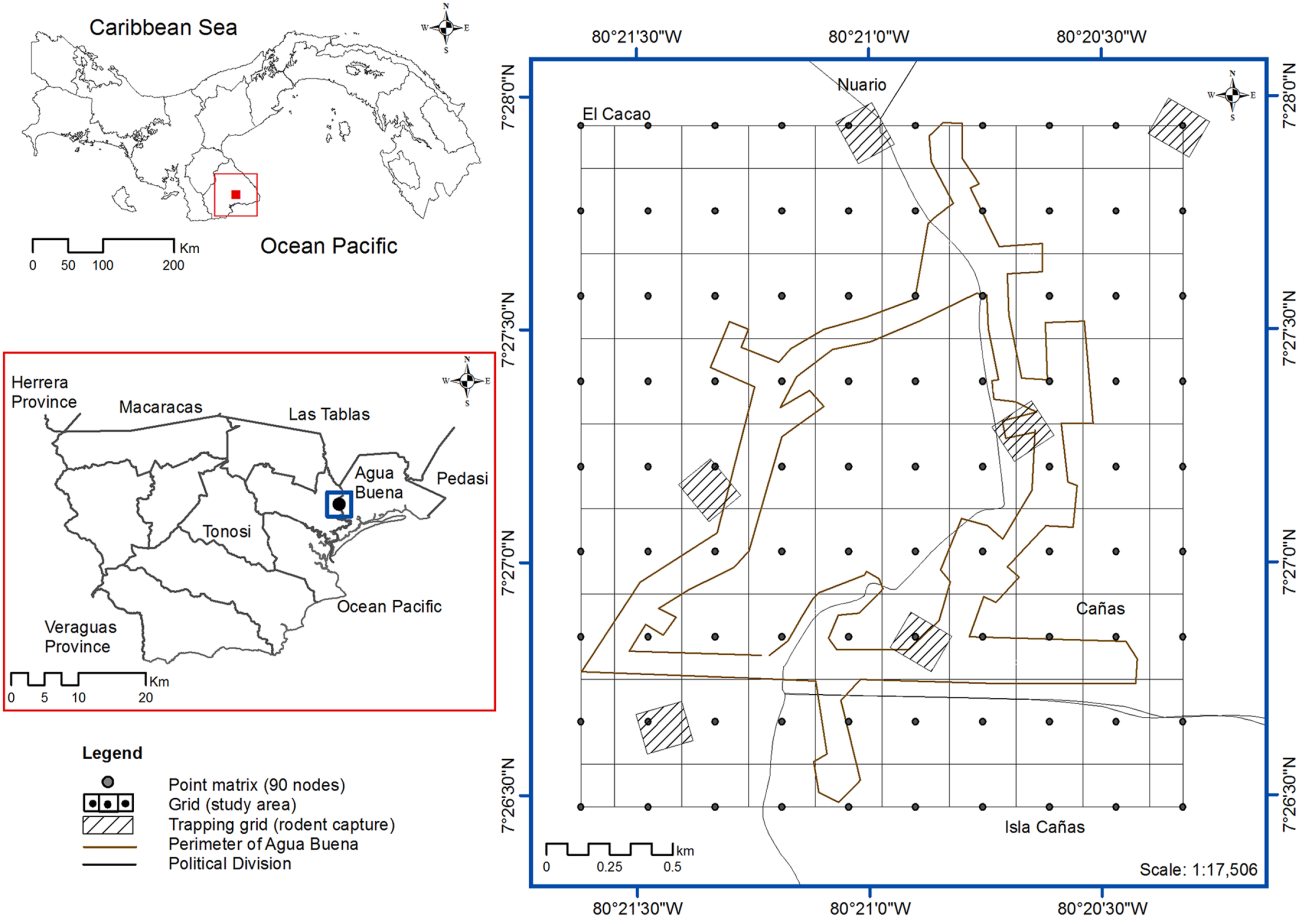
Map of study area in Agua Buena, Tonosí.

This study was carried out in the rural community of Agua Buena (peridomestic area), Tonosí County (Fig 1). Tonosí County is located at the southern coast of Azuero peninsula of Panama. Tonosí County is comprised of 1286.5 km^2^ [12] of seasonally flooded low land. Mountains separate the region from the rest of the Azuero peninsula. Located between 18 and 94 MASL (meters above sea level), the areas encompassing Agua Buena community represent approximately 0.5% (6.0 km^2^) of the Tonosí County. The 2010 census reported the population of Tonosí County as 9787 inhabitants, showing little variation when compared with the previous population census (9736 inhabitants) conducted in 2000. The main activity in Tonosí’s agroecosystem is mechanized rice production. Between 3500 and 5000 hectares per year have been cultivated in the last five years [13]. Other important cultivars planted in the Tonosí County are corn, several types of vegetables, and crops for export (e.g. melon and watermelon), which promote the economy of this region. Grass-fed livestock use 40% of the land area, which is planted with exotic gramineae for new pasture areas [11]. In addition, subsistence farming is prevalent.

The region experiences two seasons: the dry season between January and March, and the rainy season, from May to November. The months of April and December are considered as transition months [14]. The average annual precipitation for the past 12 years was 1551±1199 mm (Department of Hydrometereology of Panama).

### Rodent surveys

A mark-recapture longitudinal study was conducted in the community of Agua Buena in Tonosí, Panama, between April 2006 and December 2009. Six trapping grids representing different habitats were selected in the peridomestic area of Agua Buena community (Fig 1). Sherman traps were placed (3x3.5x9 inch, Sherman HB Inc., Tallahassee, FL) in a 10 x 10 grid with a distance of 10 meters between traps [11].

Initially, these habitats were selected to include the preferred cultivars and native vegetation, which included rice crops, corn, native pasture, introduced grasses, weeds (measuring from 10 to 200 cm in height) and a patch of secondary forest (with trees measuring 30 m high). These habitats were monitored for vegetation changes (agricultural and native vegetation composition cycle and/or status) precipitation and humans' interventions (soil preparation, planting and harvest, agrochemical fertilization and pest control, and field burning). Variations in the vegetation status, precipitation and human intervention were recorded during each sampling period during the study. Trapping grids were separated by a minimum distance of 500 m and a maximum of 3000 meters (Fig 1).

All trapping grids were georeferenced with a Global Positioning System (GPS) receiver (Garmin 60CSx) using the WGS 84 / UTM zone 17N system, and their central points (centroids) were selected. Data collection began in April 2006 and ended in December 2009. Trapping was performed on a monthly basis for four nights each month. After anesthetizing the subjects, capillary blood samples were drawn from the right retro-orbital sinus with a capillary tube containing heparin[15]. Six drops of blood were collected on filter paper (Whatman 3 MM CHR) and a numbered ear tag was placed on the subjects (National Band and Tag Co., Newport, Kentucky, USA). Morphometric measures were recorded to identify the genus and species of individuals using taxonomic keys and figures [16]. The information was recorded in field sheet format [11]. After processing, individual animals were released at their capture sites [6].

### Spatial analyses

We implemented the spatial statistical analyses in three stages. First, we generated a grid (study area) with 90 nodes of continuous information (raster format) based on estimated abundances at locations between six trapping grids by generating a continuous surface of estimated abundance using the inverse distance weighting (IDW) [17] (Fig 1). In other words, to transform the data from a finite number of samples into a continuous space in order to know the variation pattern in the area. This could be comparable to the one observed in the sample, and would help to characterize areas where information is not available. Towards this end, the Inverse Distance Weighted (IDW) method was implemented (De Smith et al., 2013), in order to interpolate, with information on the entire domain of the study, at a resolution of 336 meters. The area between the nodes was 0.09 km^2^ (266.7 x 338.7 m) and the total area of study was 6.43 km^2^ (2386.3 x 2694.1 m). Second, we examined the spatial association patterns among local populations of *O*. *fulvescens* using spatial analysis by distance indices (SADIE). The temporal structures of spatial aggregation and centers of aggregation dynamics was determined by measuring the distance to which the individuals moved, and identifying areas of hot (clusters with high rodents abundance) and cold spots (low rodents abundance)[18,19].

Then, we identified the areas of hot and cold spots using a weight matrix by the "Queen" method of second-order contiguity –i.e., in spatial autocorrelation adhere to a common definition of neighborhood relations and in the queen case considers a neighborhood of eight cells [20,21]. Spatial autocorrelation was examined using exploratory spatial data analysis by calculating Moran's I, Moran’s correlograms, and Local Indicators of Spatial Association (LISA) [20,22].

### Ethics

The study was evaluated and approved by the Institutional Animal Care and Use Committee of the Gorgas Memorial Institute for Health Studies (# 001/05 CIUCAL/ICGES, July 4, 2005); using the criteria established in the "International Guiding Principles for Biomedical Research Involving Animals developed by the Council for International Organizations of Medical Sciences (CIOMIS). The study was in accordance with Law No. 23 of January 15 1997 (Animal Welfare Assurance) of República de Panamá.

## Results

We conducted 46 sampling periods between 2006–2009, with a sampling effort of 89,449 effective trap-nights (total of traps displayed excluding closed traps, damaged or crushed traps, traps capturing other species), and 4,113 individuals of six species were captured: *Zygodontomys brevicauda* (74.4%), *Sigmodon hirsutus* (9.9%), *Oligoryzomys fulvescens* (9.1%), *Liomys adspersus* (5.8%), *Mus musculus* (0.1%) and *Oecomys concolor* (0.8%). Here, we focus on the 373 (9.1%) *Oligoryzomys fulvescens*, because of its status as a reservoir host of Choclo virus.

### Seasonal distribution

**Fig 2:**
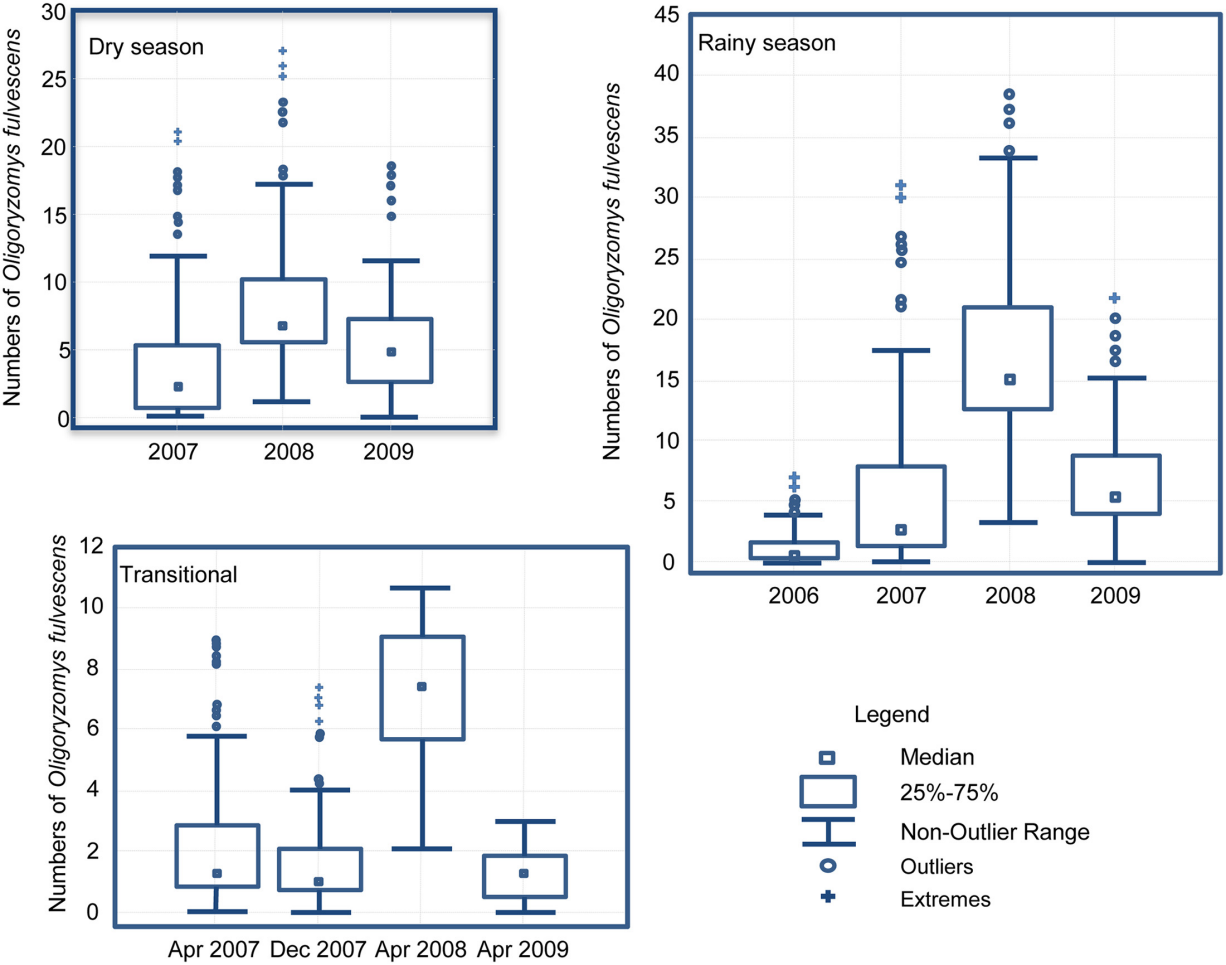
*Oligoryzomys fulvescens* distribution by season (Dry, rainy, and transitional) and year using Box-Plot in Agua Buena, Panama, April 2006-December 2009.

Sampling began in April 2006 but no *O*. *fulvescens* were captured until the beginning of the rainy season of that year and during that time fewer than 3 rodents were captured (Fig 2). Again, in December 2006, no *O*. *fulvescens* were captured, but they were captured at the sites again early in 2007, during the dry season. *O*. *fulvescens* persisted through all of 2007 and reached peak abundance in 2008 before rodents locally absent during the late transitional period of the season in 2008. Following the year of peak abundance, *O*. *fulvescens* declined but persisted until the late transitional of the season in December 2009 –when population once again became locally absent (Fig 2).

### Moran's I Correlograms

**Fig 3:**
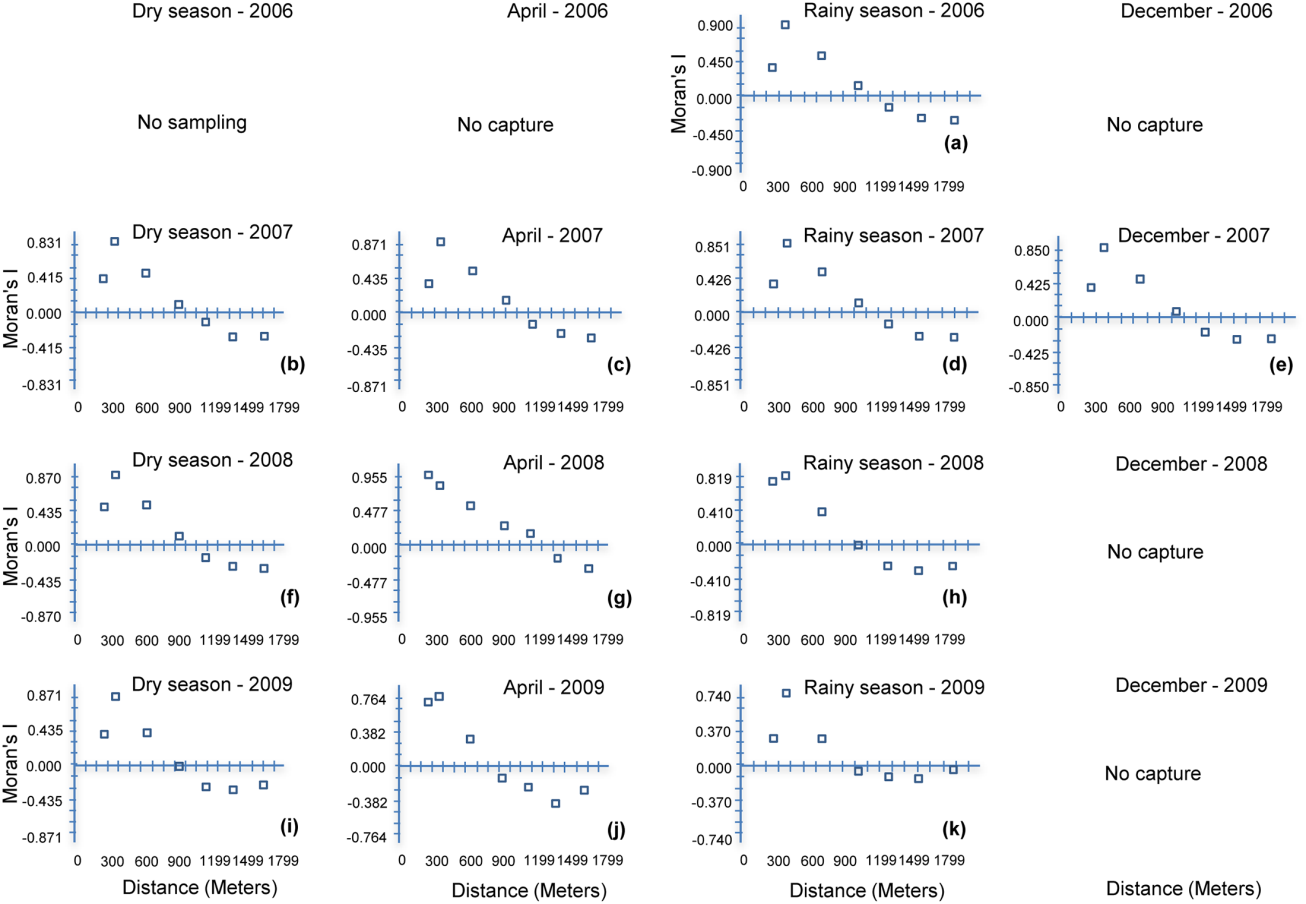
Moran’s I correlograms of *Oligoryzomys fulvescens* by season, in Agua Buena, Panama, April, 2006–December, 2009.

The local spatial abundance of *O*. *fulvescens* was spatially aggregated over relatively short distances, as characterized by Moran's I regardless of climatic periods (dry season, rainy season and transitional period of the season). This was demonstrated by strong positive spatial autocorrelations, at lag distances of 300 m–600 m (Fig 3a–3k). At distance larger than 600 m, abundances tended to be uncorrelated.

### Rodents’ spatial behavior pattern

**Fig 4:**
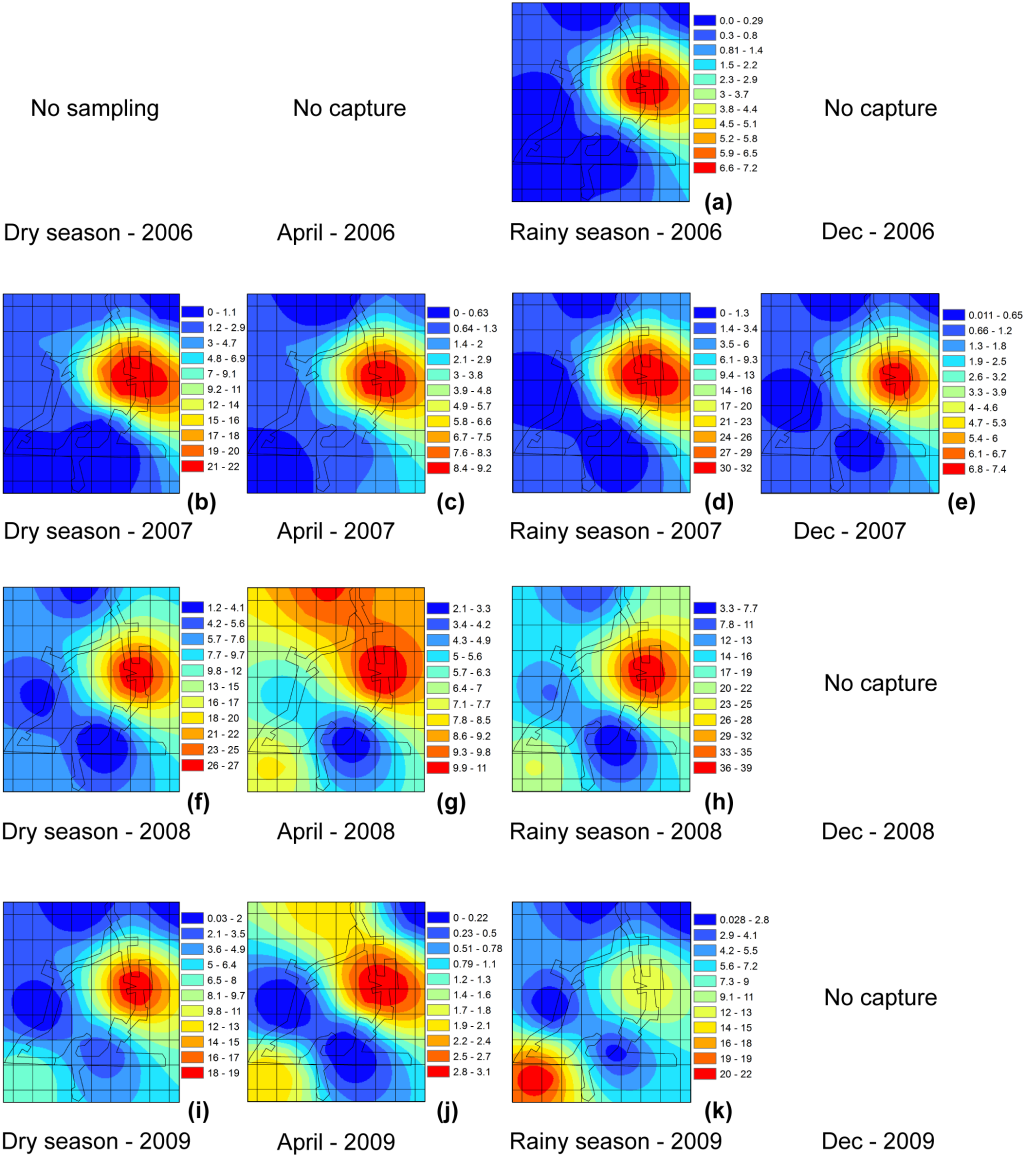
Map of the spatial distribution of *Oligoryzomys fulvescens* by season, in Agua Buena, Panama, 2006–2009.

The global spatio-temporal pattern observed for the abundance of *O*. *fulvescens* showed a shift in hot spots that changed over multiple years. Initially, when they were captured in the Agua Buena region, there was a cluster in the upper right region of the study (Fig 4) that reappeared with the species in the 2007 dry season, and persisted until the April 2008 transitional period of the season when the species became more widely distributed in the region. By the 2008 rainy season, a new hotspot emerged in the lower left quadrant of the study area, and over the next year it gradually replaced the original hot spot as the upper right area became less important for the species (Fig 4).

#### SADIE

The persistent hotspot from 2007–2008 in the upper right region of the study area and the initial persistence of low abundance clusters of *O*. *fulvescens* were confirmed by SADIE analyses. This analysis showed spatial clusters, where the values of the *Ia* index of all periods were highly significant (*Pa*<0.01) (Table 1).

**Table 1:** Summary of SADIE analyses, in Agua Buena, Panamá, 2006–2009.

Year	Season	*I*a	Pa	*vj*	*Pj*	*vi*	*Pi*
2006	April*	-	-	-	-	-	-
	Rainy	2.840	<0.0002	-2.822	0.0000	2.820	0.0000
	December*	1.463	<0.0122	-1.489	0.0119	1.323	0.0421
2007	Dry	3.066	<0.0002	-2.633	0.0000	2.696	0.0000
	April	2.526	<0.0002	-2.380	0.0000	2.271	0.0000
	Rainy	3.069	<0.0002	-2.612	0.0000	2.515	0.0000
	December	2.609	<0.0002	-2.479	0.0000	2.068	0.0000
2008	Dry	2.588	<0.0002	-2.582	0.0000	2.277	0.0000
	April	2.971	<0.0002	-2.816	0.0000	2.640	0.0000
	Rainy	2.110	<0.0002	-2.110	0.0000	1.824	0.0000
	December	-	-	-	-	-	-
2009	Dry	2.674	<0.0002	-2.557	0.0000	2.697	0.0000
	April	1.846	<0.0002	-1.849	0.0002	1.620	0.0012
	Rainy	2.692	<0.0002	-2.491	0.0000	2.115	0.0000
	December	-	-	-	-	-	-

*I*a: Index aggregation. *P*a: probability. *vj*: negative value (gap), *Pj*: probability. *vi*: positive value (clusters), *Pi*: probability.

*Corresponding to transitional month between seasons

(-) No information.

#### LISA

**Fig 5:**
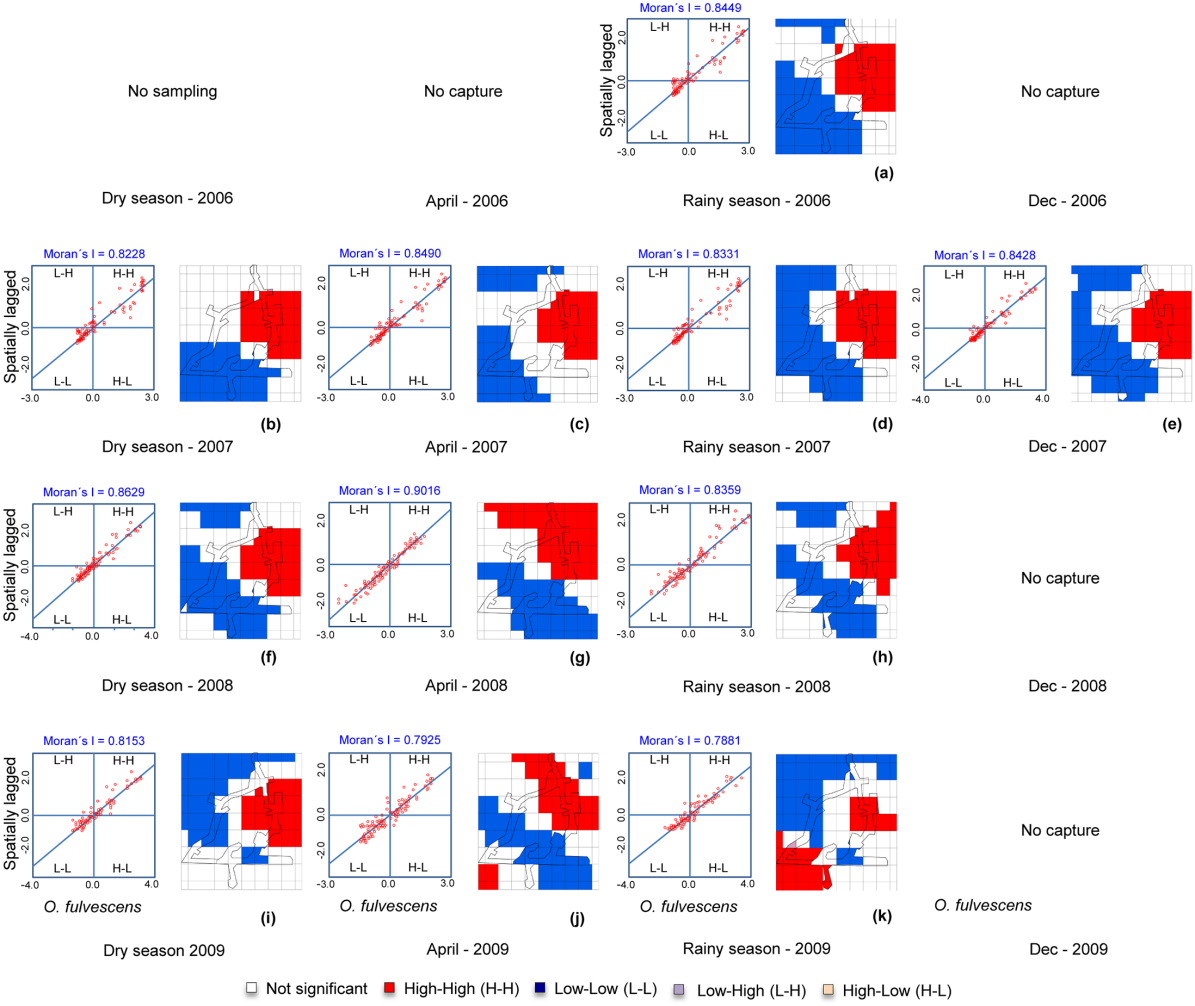
LISA distribution of *Oligoryzomys fulvescens* by season, in Agua Buena, Panama, 2006–2009.

Most of the sampling sites were associated with either high-high or low-low abundances as shown by the high concentration of points in quadrants I and III (Fig 5). There are relatively few sampling points in which high-low or low-high sampling sites were adjacent, suggesting the suitable and unsuitable sites formed contiguous patches. These patches of high or low abundance were separated by very narrow interface zones (not significant; Fig 5). The original suitable patch persisted for approximately two years until April 2009 when the second suitable patch appeared in the lower left of the region, while the original patch supported fewer *O*. *fulvescens*.

## Discussion

Here, we explore the multi-annual spatial dynamics of *O*. *fulvescens* local populations, demonstrating seasonal and yearly heterogeneity both in abundance and spatial distribution. This type of variation in zoonotic reservoir populations makes pathogen hotspot identification quite challenging. In addition, our results provide a detailed spatio-temporal analysis that characterizes the geographic extent of hot spots of rodent habitation and persistence. These hotspots are quite localized, and are prone to shifting across time.

During the four years of this study, the distribution and abundance of the *O*. *fulvescens* populations fluctuated widely both among the seasons and across years. Nearly every year, *O*. *fulvescens* were not captured during the December transitional period of the season, and the populations re-established themselves at the trapping grids at the beginning of the dry season. They increased in abundance as the rainy season progressed, and then were locally absent the following transitional period of the season. In addition to this annual pattern, there were substantial inter-annual fluctuations in abundance. The abundance of populations was low at the start of the study, in 2006, and increased in subsequent years to a peak in 2008 before declining again in 2009 (Fig 2). Both intra- and inter-annual variation in abundance of most rodent population and also of other wildlife populations is well known, in temperate regions where marked variation in temperature and day length impacts ecosystems [23–25]. However, similar observations are reported from tropical and subtropical regions but precipitation may be the primary driver of ecosystem productivity in tropical and subtropical regions [26–28].

The repeated absence of *O*. *fulvescens* at trapping sites during the transitional months of the season followed by its re-establishment suggests that the population is being maintained by repeated recolonization from somewhere off the study site and that in the absence of dispersal it may be unable to be maintained (e.g. Glass et al 2007). The repeated recolonization of the rodents of the same region of Agua Buena (Fig 4) for several years suggests that some aspects of the environment in this area was key to the establishment of the population [11]. However, these environmental components were not permanent and by mid-2008 there was the beginning of the establishment of *O*. *fulvescens* populations to the southern portion of the study area and the original population cluster in the northwest began to decline. The geographic shift in abundance was nearly complete by the end of the study (Fig 4). Some regions of the total study area appeared to remain unsuitable for *O*. *fulvescens* throughout the entire study. These regions of low abundance in the northwest and southeast isolated the two smaller regions of high abundance of *O*. *fulvescens*. We detected highly significant autocorrelations in population abundances as measured by Moran’s I (Fig 3), which we use as a proxy for suitability of habitat. Across all sites, there was a trend of moderate negative correlation, suggesting only a portion of the study area was suitable for *O*. *fulvescens* habitation. This was most evident during the dry and transitional times and least evident during the rainy season (Fig 3)–suggesting that factors related to vegetation may influence the seasonal abundance/distribution of this rodent.

In addition to the spatial progression of suitable habitat by 2008–2009, the geographic extents of the local populations of *O*. *fulvescens* was concentrated. The spatial autocorrelation analyses (Fig 3), and SADIE (Fig 5) show that autocorrelation in captures and sites with high abundances were clustered over relatively short distances (< 1km). Beyond these distances either captures were not correlated or had reached the extent of the unsuitable habitat and were negatively correlated. The interface between sites with high-high and low-low (Fig 5) abundances typically occurred within a short distance. This may imply that local populations very rapidly saturate the suitable habitats before occupying an ecotone of marginal conditions. This rapid demographic response may be one characteristic of an efficient (“good”) reservoir species [3] and makes a reservoir/pathogen emerge suddenly, increasing the risk of disease outbreak ‘every-or-anywhere’.

Our results provide an important characterization of the challenges in monitoring or predicting zoonotic disease outbreaks at the level of reservoir populations. These populations are subject to fluctuations over time (both within and among years), and the populations themselves are geographically restricted, even within their range. Suitable habitat may change, but these dynamics on the landscape level is poorly understood. Despite local persistence in a well-defined region for several years, the population may undergo shifts and establishment in new areas. Future studies should clearly delineate what environmental factors and seasonal cues may be crucial to habitat shifts by zoonotic hosts.

## Supporting Information

S1 Database Database of *Oligoryzomys fulvescens* by season and precipitation in Agua Buena. (XLSX)
